# Improved biomechanics in experimental chronic rotator cuff repair after shockwaves is not reflected by bone microarchitecture

**DOI:** 10.1371/journal.pone.0262294

**Published:** 2022-01-05

**Authors:** Xaver Feichtinger, Patrick Heimel, Stefan Tangl, Claudia Keibl, Sylvia Nürnberger, Jakob Emanuel Schanda, David Hercher, Roland Kocijan, Heinz Redl, Johannes Grillari, Christian Fialka, Rainer Mittermayr

**Affiliations:** 1 Ludwig Boltzmann Institute for Experimental and Clinical Traumatology, Vienna, Austria; 2 AUVA Trauma Center Vienna—Meidling, Vienna, Austria; 3 Department of Orthopaedic Surgery II, Herz-Jesu Krankenhaus, Vienna, Austria; 4 Austrian Cluster for Tissue Regeneration, Vienna, Austria; 5 Karl Donath Laboratory for Hard Tissue and Biomaterial Research, Department of Oral Surgery, University Clinic of Dentistry, Medical University of Vienna, Vienna, Austria; 6 Division of Trauma-Surgery, Department of Orthopaedics and Trauma-Surgery, Medical University of Vienna, Vienna, Austria; 7 Ludwig Boltzmann Institute of Osteology, 1st Medical Department at Hanusch Hospital, Vienna, Austria; 8 Center for the Musculoskeletal System, Medical Faculty, Sigmund Freud University, Vienna, Austria; Szegedi Tudomanyegyetem, HUNGARY

## Abstract

**Purpose:**

The aim of this study was to investigate the effect of extracorporeal shockwave therapy (ESWT) on bone microstructure as well as the bone-tendon-interface and the musculo-tendinous transition zone to explain the previously shown improved biomechanics in a degenerative rotator cuff tear animal model. This study hypothesized that biomechanical improvements related to ESWT are a result of improved bone microstructure and muscle tendon properties.

**Methods:**

In this controlled laboratory study unilateral supraspinatus (SSP) tendon detachment was performed in 48 male Sprague-Dawley rats. After a degeneration period of three weeks, SSP tendon was reconstructed transosseously. Rats were randomly assigned into three groups (n = 16 per group): control (noSW); intraoperative shockwave treatment (IntraSW); intra- and postoperative shockwave treatment (IntraPostSW). Eight weeks after SSP repair, all rats were sacrificed and underwent bone microstructure analysis as well as histological and immunohistochemical analyses.

**Results:**

With exception of cortical porosity at the tendon area, bone microstructure analyses revealed no significant differences between the three study groups regarding cortical and trabecular bone parameters. Cortical Porosity at the Tendon Area was lowest in the IntraPostSW (p≤0.05) group. Histological analyses showed well-regenerated muscle and tendon structures in all groups. Immunohistochemistry detected augmented angiogenesis at the musculo-tendinous transition zone in both shockwave groups indicated by CD31 positive stained blood vessels.

**Conclusion:**

In conclusion, bone microarchitecture changes are not responsible for previously described improved biomechanical results after shockwave treatment in rotator cuff repair in rodents. Immunohistochemical analysis showed neovascularization at the musculo-tendinous transition zone within ESWT-treated animals. Further studies focusing on neovascularization at the musculo-tendinous transition zone are necessary to explain the enhanced biomechanical and functional properties observed previously.

**Clinical relevance:**

In patients treated with a double-row SSP tendon repair, an improvement in healing through ESWT, especially in this area, could prevent a failure of the medial row, which is considered a constantly observed tear pattern.

## Introduction

Depending on tear size, healing failure and re-rupture rates after rotator cuff repair are reported from 20% up to 94% [1, 2]. Bony changes as well as degenerative tendon structure including but not limited to loss of tendon organization seem to be important reasons [3, 4]. Osseous rarefaction in the humeral head in patients suffering from chronic rotator cuff tears were shown earlier [5]. Bony deteriorations, such as osteoporosis, were described to be an important risk factor of healing failure after rotator cuff repair [6]. Chung et al. showed, that especially the decrease in Bone Mineral Density (BMD) and fatty infiltration of muscle and tendon degeneration in chronic tendon ruptures have a direct influence on postoperative healing [6]. Also structural bone changes, detected by high resolution quantitative computed tomography, have been shown to be associated with rotator cuff tears [7]. Degenerative changes of muscles and tendons structures such as intramuscular and myocellular fat infiltration, atrophy, fibrosis and loss of tendon structure also have an important influence on the healing rate after rotator cuff repair [8, 9].

Extracorporeal shockwave therapy (ESWT) has shown a positive influence on tissue regeneration in experimental studies and clinical trials [10, 11]. Tendon regeneration with modulation of cell proliferation, decreased expression of inflammation markers as well as improved muscle regeneration seem to be important key mechanisms of ESWT [12–14]. Especially Vascular Endothelial Growth Factor, known to induce angio- as well as lymphangiogenesis [15], cell proliferation via the extracellular signal-regulated kinase 1/2 pathway [13] as well as inflammatory modulation via Toll-Like Receptor 3 [16]. The positive influence on bone metabolism and improved healing has also been described several times and is routinely used in the clinic for various bone pathologies [17–19]. Increased FGF-2 production by osteoblasts stimulated using ESWT was reported to improve bone formation as well as bone healing [20]. However, studies investigating the effect and mechanisms of ESWT in rotator cuff pathologies are rare. Recently, we were able to show the effect of ESWT in an experimental study very clearly. Substantial improvement of biomechanical properties as well as shoulder function was shown using ESWT after rotator cuff repair in rodents with degenerative tendon tears [21].

The aim of this study was to investigate the effect of ESWT on bone microstructure as well as the bone-tendon-interface and the musculo-tendinous transition zone to explain the previously shown improved biomechanics in a degenerative rotator cuff tear animal model.

It was hypothesized, that proved biomechanical improvements related to ESWT are a result of ameliorated bone microstructure as well as favorable changes in the bone-tendon-muscle interfaces after repair of chronic rotator cuff ruptures in a rat model.

## Material and methods

**Fig 1:**
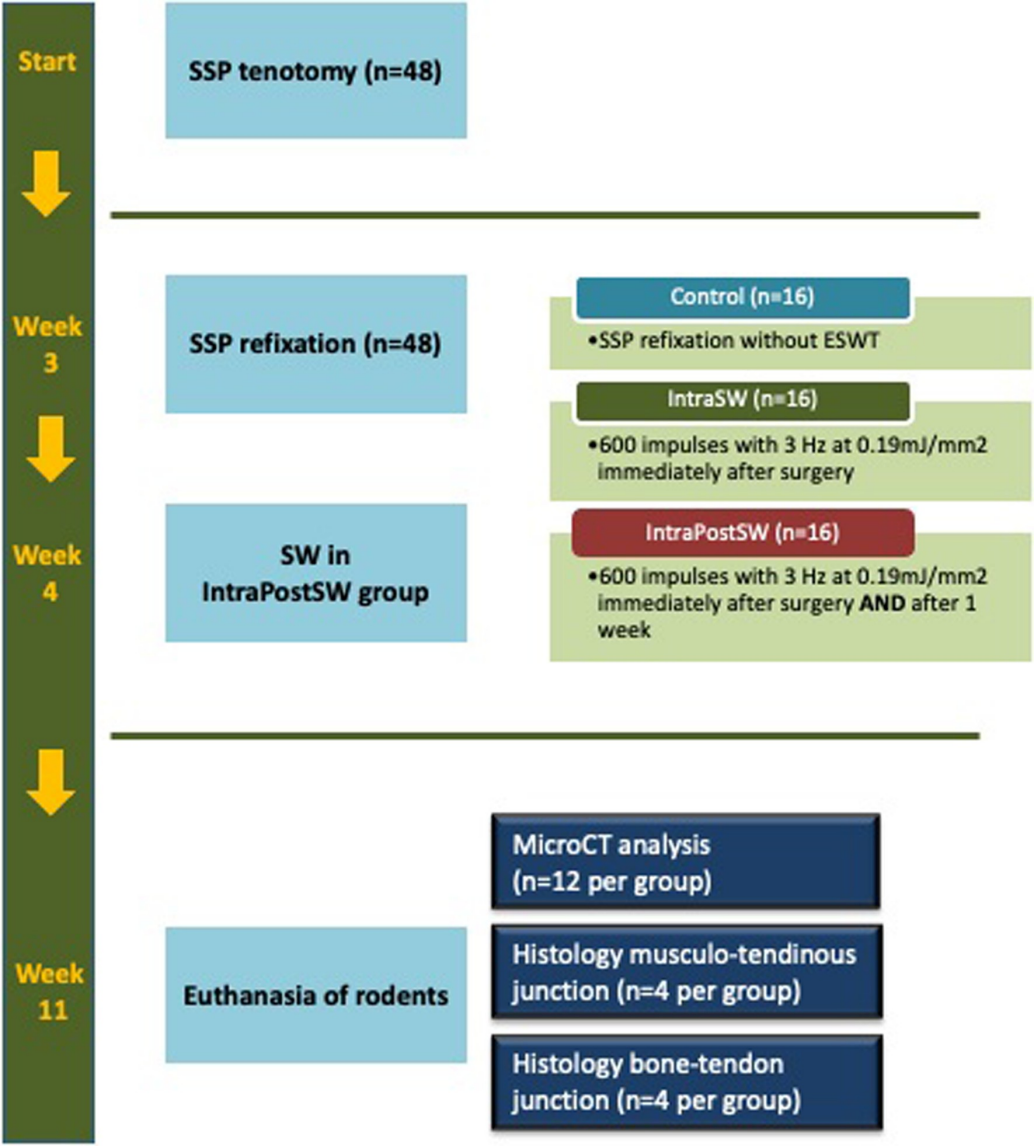
Schematic representation of the study design. SSP: supraspinatus; SW: shockwave therapy; IntraSW: intraoperative SW; IntraPostSW, intra- and postoperative SW.

**Fig 2:**
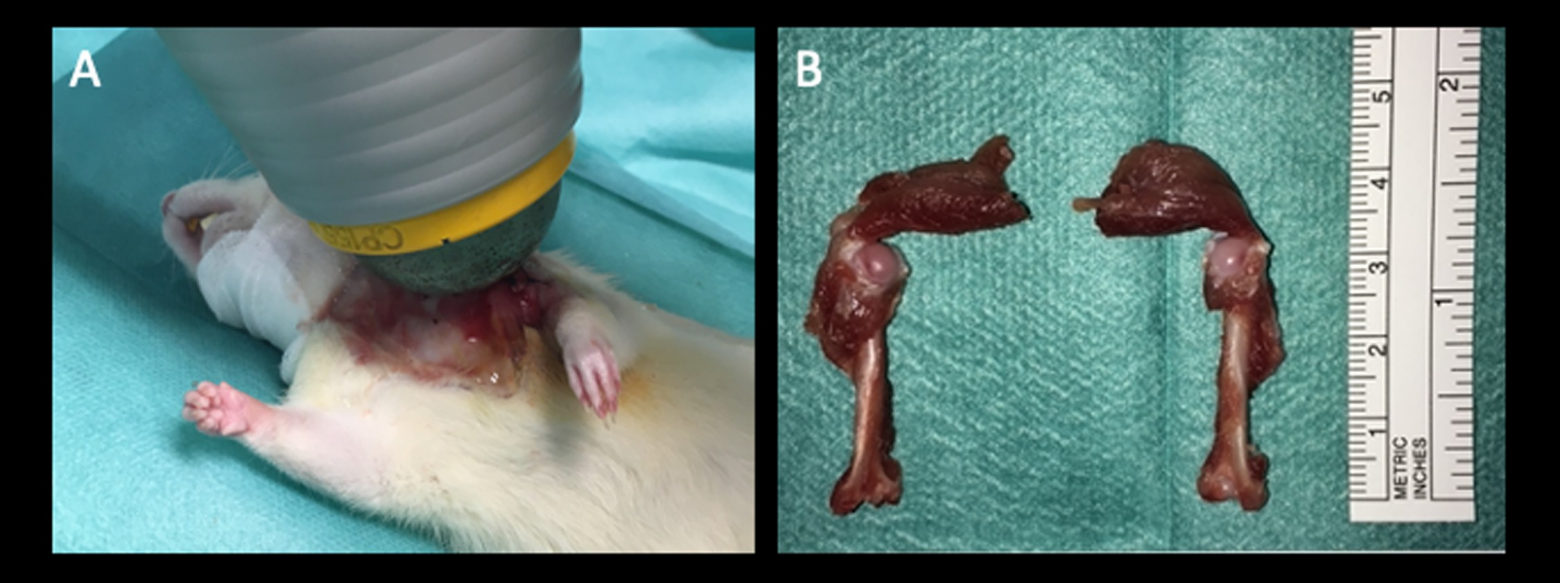
A: Shockwave treatment subsequently after tendon repair in IntraSW and IntraPostSW group. B: Preparation of the specimens immediately after euthanasia (left: operated side, right: non-operated side).

The study was approved by the local Institutional Animal Care and Use Committee (Municipal department 58 of the City of Vienna; No. 504113/2016/16). All methods were carried out in accordance with relevant guidelines and regulations. 48 male Sprague-Dawley rats (400–410 g) were used for this study. Rats were housed in cages pairwise, individually identified by a tail-mark, in a temperature- and light-controlled room. Access to food and water was provided ad libitum and continuous weight control was performed once a week. All rats were randomly assigned to one of three groups (n = 16 per group): control/noSW; intraoperative shockwave group (IntraSW); intra- and postoperative shockwave group (IntraPostSW) (Fig 1). At time point zero, all rats underwent unilateral supraspinatus (SSP) tenotomy of the left shoulder as previously reported [22]. After an anterolateral skin incision, the deltoid muscle was split in fiber direction. Surgical dissection of the SSP tendon and sharp detachment of its bony insertion at the humeral head was carried out. Due to adhesion and scar tissue formation the SSP tendon was reinforced with a suture and left subcutaneously in order to facilitate identification in the follow-up surgery [21]. Then the deltoid muscle was closed and skin was sutured. Three weeks after the initial surgery, SSP repair was conducted in all rats. Through the same skin incision, the deltoid muscle was sharply split and the SSP tendon insertion area was gently debrided. The SSP tendon was identified and a modified Mason-Allen stitch using a Prolene 5–0 suture, (Johnson & Johnson, Ethicon Inc., New Jersey, US) for tendon refixation was performed. For transosseous refixation, a bone tunnel in anteroposterior direction close to the greater tuberosity of the humerus was drilled. By passing the suture through the tunnel the tendon was readapted and secured at the anatomic insertion area [21]. Closure of the deltoid muscle and skin closure was performed equally to the first operation. Immediately after skin closure, the IntraSW and IntraPostSW group received percutaneous electrohydraulic generated ESWT (600 impulses; 0.19 mJ/mm2 energy flux density, 3 Hz (DermaGold; Tissue Regeneration Technologies, LLC; manufactured by MTS Europe GmbH)) with focus on the SSP tendon and the humeral head under anesthesia (Fig 2A). All surgical procedures were performed in general anesthesia (inhalational anesthesia with a mixture of isoflurane/oxygen) by a veterinarian and under subcutaneous (buprenorphine) and per os (meloxicam) analgesia. Fluid loss was substituted by subcutaneous acetat fluid. Free cage activity with enriched environment for recovery was allowed to all rats. Rats were monitored regularly by a veterinarian and fluid substitution as well as postsurgical analgesia was performed. One week after repair surgery, the IntraPostSW group received a second shockwave treatment in the same intensity as the first therapy. Eight weeks after repair, all animals were sacrificed under deep anesthesia by an overdose of thiopental intracardially. Immediately after euthanasia, the humerus of both sides were carefully exarticulated (Fig 2B). The SSP tendon was carefully prepared. The remaining rotator cuff was removed. One rat in the control group dedicated for histological analyses died after the repair surgery because of perioperative anesthesiologic complications. Another rat of the IntraPostSW group intended for histological analyses was excluded due to joint infection.

### Micro-Computed Tomography (microCT)

For microCT analyses 36 rats were used (12 per group). Both sides (operated left and non-operated right) were scanned immediately after exarticulation [23]. Scanning and segmentation was performed by a blinded examiner. Subsequently, all specimens were placed in 15 ml-centrifuge tubes without any additional substances. MicroCT scans (μCT 50, SCANCO Medical AG, Brüttisellen, Switzerland) were performed at 90 kVp, 200 μA, 0.5 mm Al Filter with 1000 Projections per 180° integrated for 500 ms with a Field of View of 20.48 mm and reconstructed to a resolution of 10 μm. Calibration of the scanned images was conducted with the SCANCO calibration phantom. The orientation of the humerus was standardized using the open-source platform Fiji for biological-image analysis [24]. All specimens were then aligned along the z-axis and rotated along that axis to the same orientation [24, 25]. The untreated right humerus was mirrored and rigidly registered to the left humerus using Amira™ with the affine registration tool (version 6.1.1, Zuse Institute Berlin, Thermo Fisher Scientific, Waltham, USA). The Definiens Developer XD™ (version 2.1.1, Definiens Inc., Cambridge, USA) was used for the segmentation of the scans. The bone tunnel at the tendon insertion area (480 μm diameter), which was drilled for transosseous refixation, and a perifocal area around this tunnel (160 μm) were excluded from calculations. The position of the tunnel was calculated by manually selecting approximately 5–10 points in the center of the remaining tunnel and fitting the central axis to the selected points by optimizing squared distances. The tunnel was copied from the treated to the registered untreated side to exclude the same region. The growth plate was segmented by manually drawing points along the growth plate on several slices and interpolating between them. Where the interpolation was not within the growth plate, additional points and slice positions were added to match the actual geometry of the growth plate more closely. The resulting regions of interest in the condyle extended across approximately 200–300 slices. An appropriate threshold was selected which was identical for all samples. Cortical and trabecular bone was separated using a combination of surface tension constrained region growing from outside the sample and a local bone volume density requirement which separates the dense outer layer of cortical bone from the less dense interior trabecular structures. The bone directly adjacent to the growth plate was excluded from measurements using the same procedure. The humerus was divided into four regions of interest (ROI): Cortex Articular Surface Area, Trabecular Bone Articular Surface Area, Cortex Tendon Area, Trabecular Bone Tendon Area. Thereby, bone-cartilage interface marked the separation of Articular Surface Area (ASA) and Tendon Area (TA). Each segmented ROI was exported as an image stack and measured using Fiji and the BoneJ plugin [26]. Trabecular microstructure parameters, including trabecular bone volume fraction (BV/TV, %), mean trabecular spacing (Tb.Sp mean, μm), and mean trabecular thickness (Tb.Th mean, μm) were analyzed. Cortical parameters including cortical porosity (Ct.Po, %), mean cortical thickness (Ct.Th mean, μm), and mean cortical pore diameter (PoreDM mean, μm) were examined. For calculation, a ratio of the operated to the non-operated side for each parameter was created.

### Histological and immunohistochemical analysis of musculo-tendinous transition zone

As mentioned above, 2 rats were excluded from the histological and immunohistochemical analysis. After euthanasia of the remaining 10 rats the SSP muscle-tendon transition zone of both shoulders (operated left and non-operated right) was used for histological and immunohistochemical analyses. Three examiners (X.F., S.N., R.M.) blinded to the study group allocation performed the evaluations. The SSP tendon was cut at a distance of 7 mm from tendon insertion area and another cut was performed at 20 mm distance. The tendon and the musculo-tendinous transition zone were fixed in 4% buffered formaldehyde solution for 24 hours. Subsequently, the samples were washed with tap water and shifted into 50% ethanol solution for 1 hour. Then they were stored in a 70% ethanol solution [27]. After embedding in paraffin wax, sections of 4 μm thickness were performed (with exception of hematoxylin and eosin (HE) staining at a 3 μm thickness). Staining with Martius, Scarlet and Blue (MSB) for collagen and fibrin as well as HE was conducted according to standard protocols [27]. Scanning and evaluating was performed using a light microscope (Axioplan2 imaging, Carl Zeiss Microscopy GmbH, Jena, Deutschland; Olympus BX61VS, Olympus Corporation, Tokyo, Japan). Immunohistochemical staining was carried out according to standard procedures using a monoclonal and a polyclonal antibody against neurofilament (NF) proteins (Dako, Santa Clara, USA; Immunologic, Duiven, NL) for nerve tissue imaging, two polyclonal antibodies against CD31 (Thermo Fisher Scientific, Waltham, USA; Immunologic, Duiven, NL), and two polyclonal antibodies against collagen III (Abcam, Cambridge, USA; Dako, Santa Clara, USA) and against collagen I (Abcam, Cambridge, USA; Immunologic, Duiven, NL) for tendon, muscle and scar tissue visualization [27–32]. The musculo-tendinous transition zone was defined as primary region of interest. Two standardized regions per sample (675 x 535 μm) at the musculo-tendinous transition zone were chosen and used for processing with a light microscope at a magnification of 20 and AxioVision microscope software (AxioVision^®^, version 3.1., Carl-Zeiss AG, Oberkochen, Germany). Evaluation of immunohistochemical samples was performed with ImageJ (version 1.51s, NIH, USA) [33].

### Histological analysis of bone-tendon transition zone

To evaluate bone-tendon healing and regeneration, hard tissue histology was performed in 10 rats. Both sides (operated left and non-operated right) were subjected to a qualitative histological analysis. Using a diamond saw, the distal third of the humerus was dissected to protect the region of interest and the SSP tendon was severed at 7 mm distance from tendon insertion area as previously mentioned. The bone-tendon specimens were fixed in 4% buffered formaldehyde solution at 4°C for 6 weeks. Undecalcified thin ground sections and Lévai-Laczkó staining were performed as described previously [34, 35]. To achieve a representative transection through the bony insertion site of the SSP tendon, slices were oriented parallel to the longitudinal axis of the humerus. Digital images with a resolution of 0.32078 μm per pixel were produced with an Olympus BX61VS scanning microscope and Olympus dotSlide 2.4 digital virtual system (Olympus^®^, Tokyo, Japan).

### Statistical analyses

A power analyses was performed with the primary outcome parameter BV/TV in microCT analysis based on a previous study investigating the effect of ESWT on BV/TV in rats [36]. With these estimations a power of 0.80 is achieved (α = .05) with 12 specimens per group. Testing for normal distribution was performed for microCT analyses using the D’Agostino & Pearson omnibus normality test. In cases of no normal distribution, the Kruskal-Wallis test and Dunn’s multiple comparisons test were conducted. In the case of a normal distribution, one-way ANOVA and Tukey’s/Sidak’s multiple comparisons tests were performed. For calculation, a ratio of the operated to the non-operated side for each parameter was created. GraphPad Prism version 6.00 (GraphPad Software, La Jolla, California, USA, www.graphpad.com) was used for statistical calculations.

## Results

Macroscopically, the SSP muscle and tendon structure showed no differences between the different groups. No suture disruptions or gap formations at the tendon insertion area were observed.

### MicroCT

**Fig 3:**
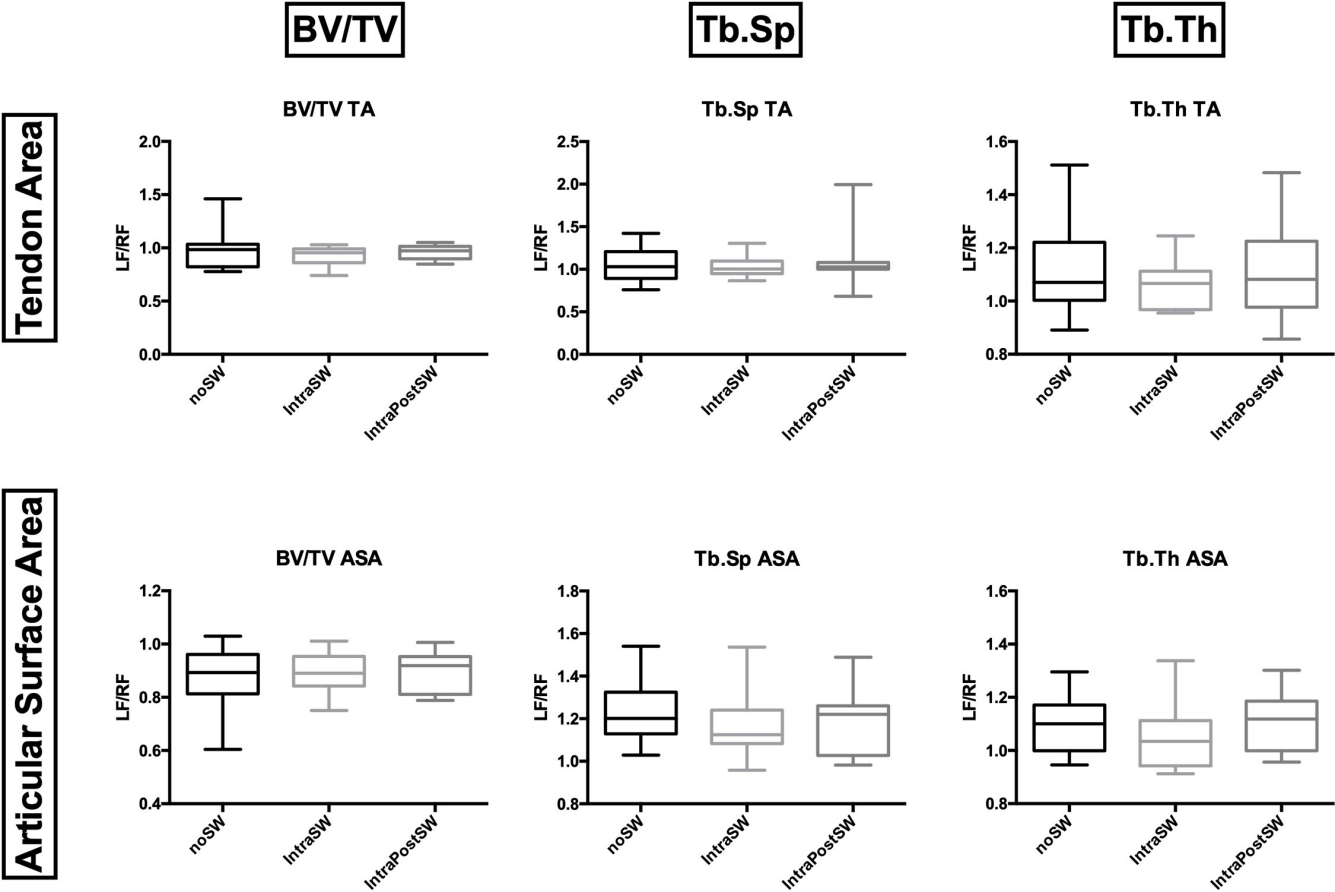
Box-and-whisker plots showing trabecular bone parameters: Bone Volume/Tissue Volume (BV/TV), Trabecular Spacing (Tb.Sp), Trabecular Thickness (Tb.Th). TA: Tendon Area, ASA: Articular Surface Area.

**Fig 4:**
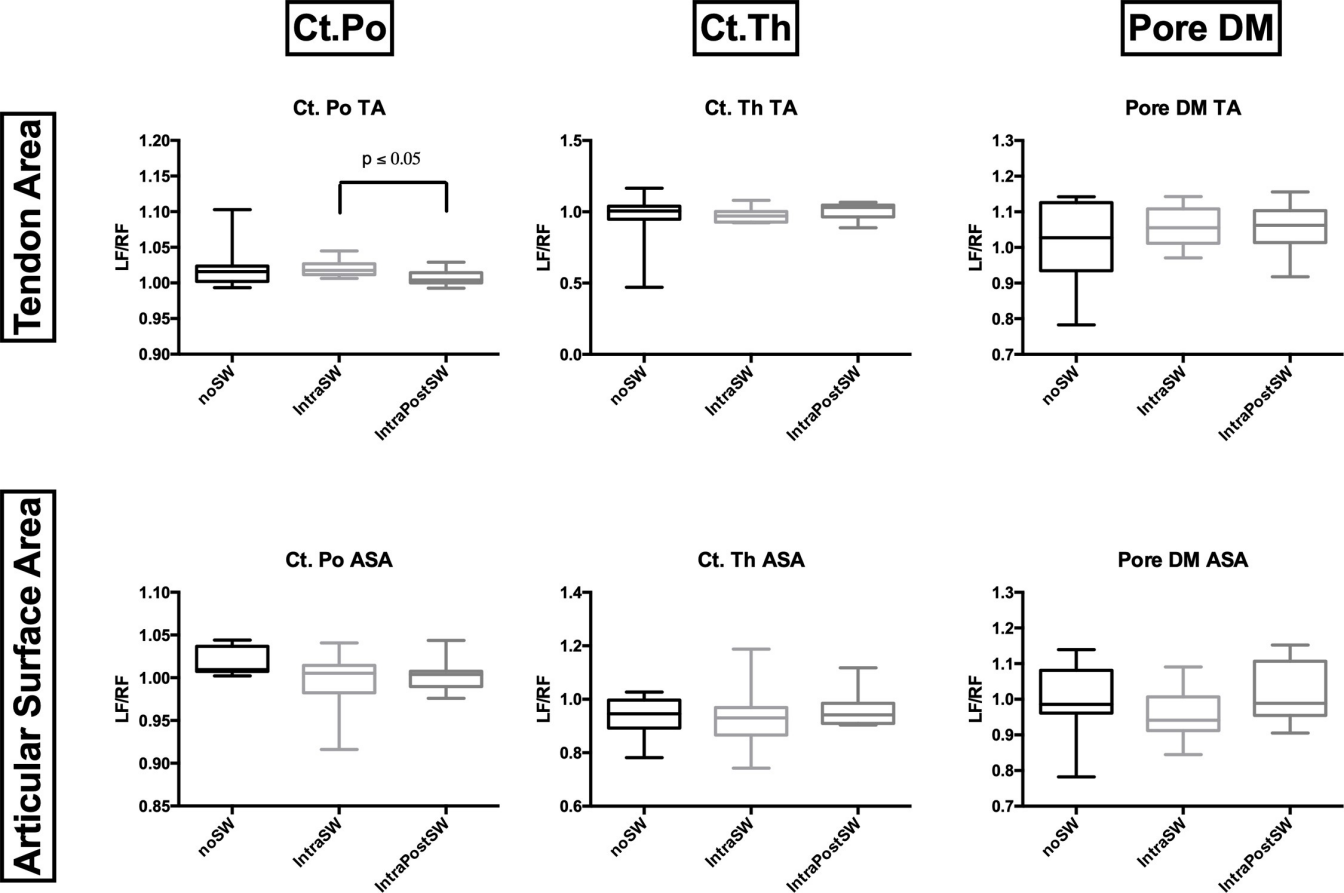
Box-and-whisker plots showing cortical bone parameters: Cortical Porosity (Ct. Po), Cortical Thickness (Ct. Th), Pore Diameter (Pore DM). TA: Tendon Area, ASA: Articular Surface Area.

No significant differences were observed in trabecular bone parameters between the three study groups (Fig 3). Cortical Bone assessment did not show any significant differences, excluding Ct. Po at the Tendon Area. The IntraSW group had a significantly higher (p ≤ 0.05) Ct. Po (%) in the Tendon Area than the IntraPostSW group (Fig 4). A higher Ct. Po was also observed in the Control Group, but because of the high variance, the differences did not reach significance.

### Histology/immunohistochemistry–musculo-tendinous transition zone

**Fig 5:**
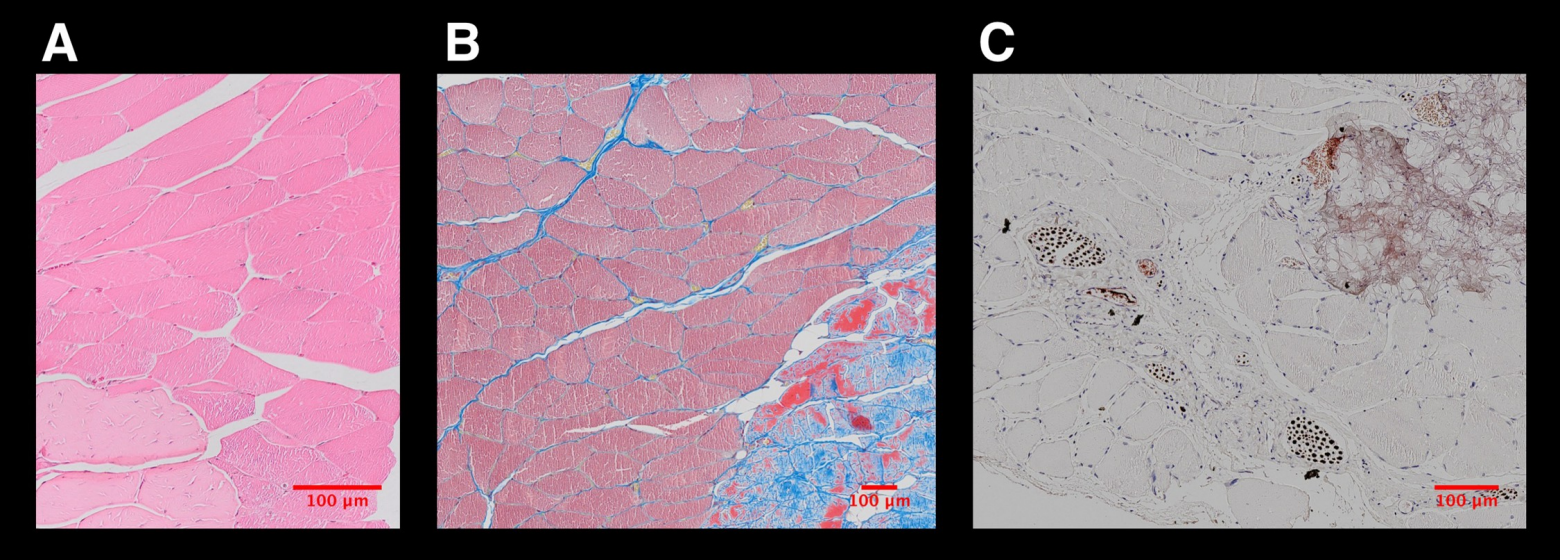
A: Hematoxylin and Eosin (H&E), B: Martius, Scarlet and Blue (MSB), and C: Neurofilament (NF) stained sections of a musculo-tendinous transition zone.

**Fig 6:**
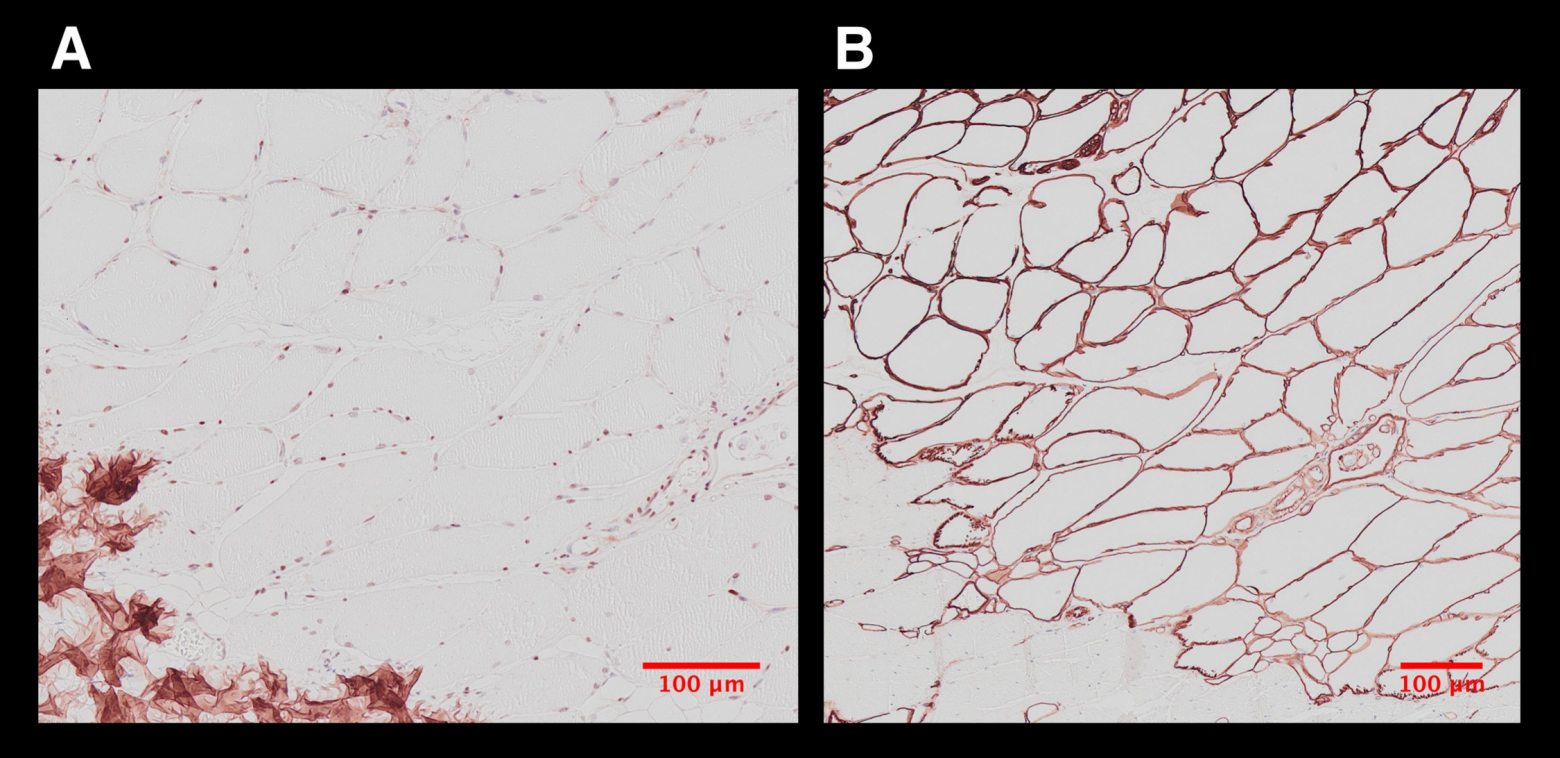
A: Collagen I and B: Collagen III stained sections of a musculo-tendinous transition zone.

**Fig 7:**
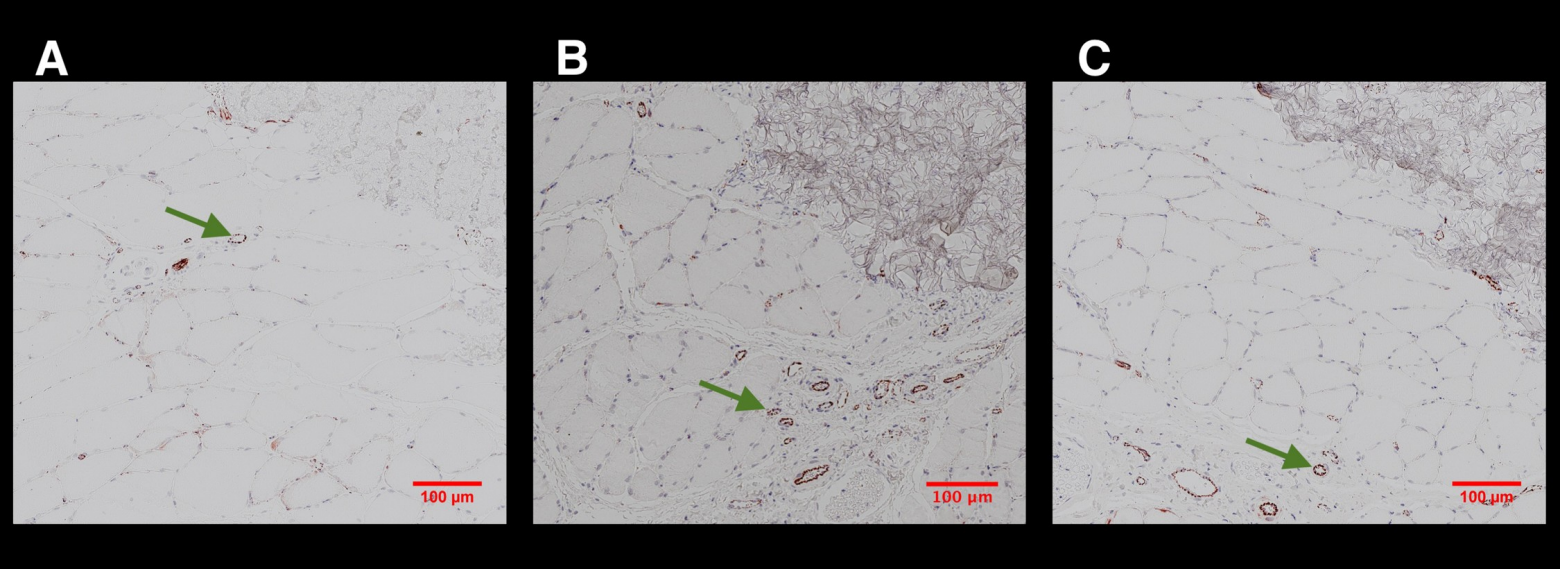
CD31 staining of musculo-tendinous transition zone: A: Control Group; B: IntraSW Group; C: IntraPostSW Group. Green Arrows are marking blood vessels.

HE and MSB staining demonstrated a regular tendon and muscle quality in all samples (Fig 5A and 5B). No differences were detected between the groups and their contralateral sides. Collagen III and Collagen I staining in muscle, tendon, and scar tissue revealed no differences among the study groups (Fig 6A and 6B). None of the three study groups showed noticeable defects or scar tissue production compared with the non-operated contralateral side. NF staining showed clearly visible nerve structures. In all study groups, nerve structures could be located, and no differences between the three groups and their contralateral sides were discernible (Fig 5C). Evaluating CD31 stained samples, a higher density of blood vessels was recognized in ESWT treated groups (IntraSW (Fig 7B) > IntraPostSW (Fig 7C) in comparison to the control group (Fig 7A).

### Hard tissue histology–bone-tendon transition zone

**Fig 8:**
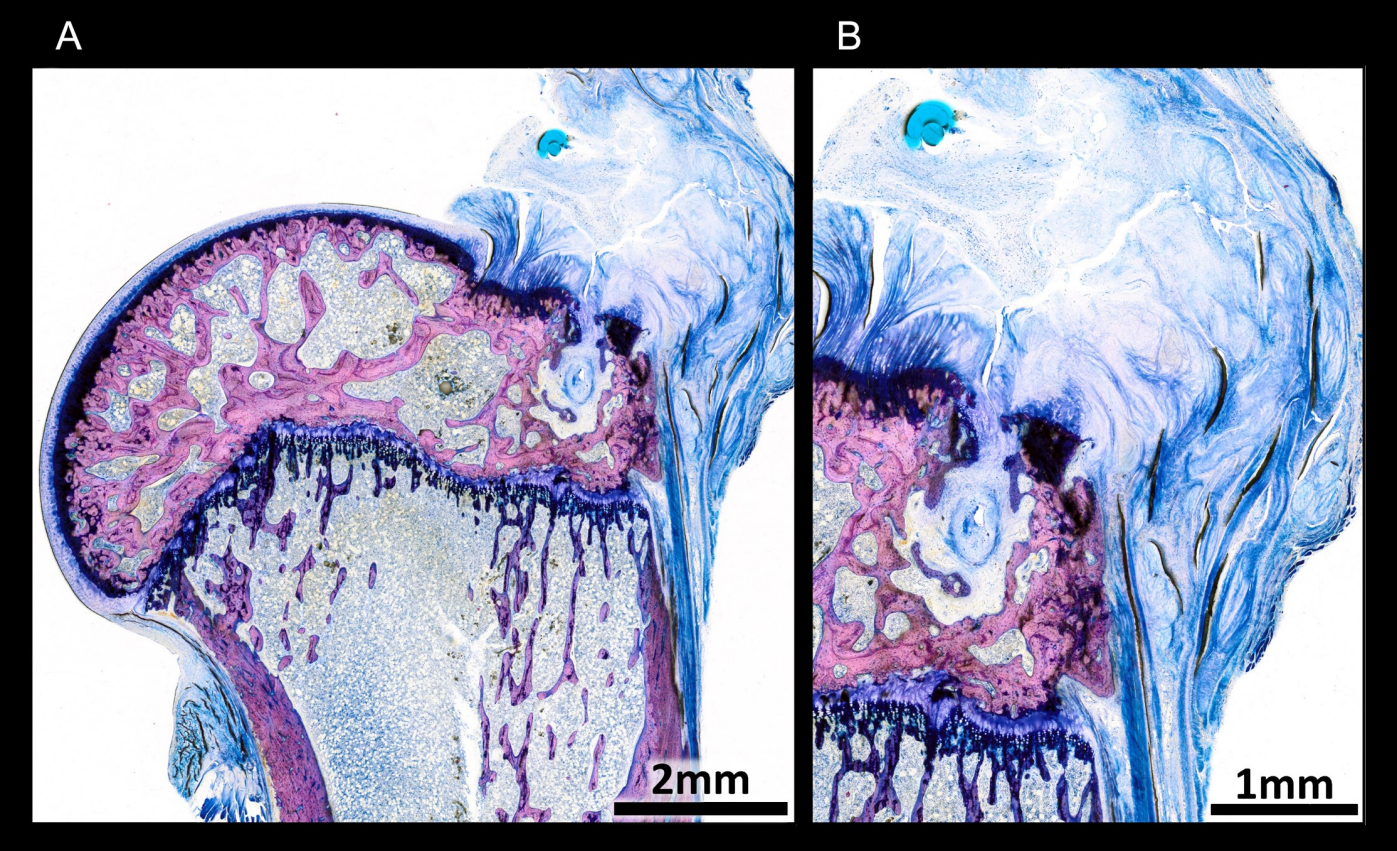
Undecalcified thin ground section and Lévai-Laczkó staining of bone-tendon transition zone.

The operated shoulders showed that the stump of the tendon that had been cut close to the bone surface was still visible, the connection between the ruptured tendon and the humerus being located on the lateral side (Fig 8). Only granulation tissue could be observed between the stump and the tendon brought in proximity to it by the suture in place. Neither the zone of calcified fibrocartilage of the former enthesis nor the lamellar bone tissue immediately underlying it, showed any differences in histological or cellular structure between groups. No signs of increased resorption or bone remodeling could be detected underneath the enthesis in animals treated with shock waves. The structure of cancellous bone in the region of the epiphysis that had not been affected by the drilling of the tunnel for tendon refixation was similar in all study groups.

## Discussion

This study aimed to evaluate the effects of ESWT on bone microstructure as well as the bone-tendon-interface and musculo-tendinous transition zone in a chronic rodent rotator cuff tear model. Bone microarchitecture after a chronic rupture was not affected by shock wave treatment after tendon repair. However, immunohistochemical analysis showed neovascularization at the musculo-tendinous transition zone in both ESWT groups which may be explaining the enhanced biomechanical properties observed previously.

Due to high failure rates in repair of chronic rotator cuff tears, techniques for improvement are of high clinical relevance [4]. Studies investigating shockwave treatment in tendon regeneration are rare. A study investigating radial pressure waves after rotator cuff repair was not able to find any improvements [37]. However, due to physical attributes, radial pressure waves are differing significantly from shockwaves and do not meet the benefits of focused ESWT [11]. Brañes et al. presented improved neovascularization and neolymphangiogenesis after rotator cuff repair in ESWT treated patients [38]. Recently, clear biomechanical and functional improvements in ESWT treated male Sprague-Dawley rats after repair of chronic rotator cuff tears have been shown [21]. Gene expression analysis showed no significant differences between the groups. A trend towards a more regenerative healing in ESWT groups in contrast to a more scar-mediated process in the control group was shown by TGF-β1/TGF-β3 ratio measurements [21].

Many studies have indicated the beneficial effect of ESWT on tissue regeneration in experimental studies and clinical trials [10, 39]. Thereby, stimulation of growth factors as well as modulation of cell proliferation and inflammation processes seem to play important key roles and ESWT was described to have a beneficial effect on bone, tendon, and muscle structures.

In this study, bone microarchitecture assessment was performed according to earlier publications and established bone structure parameters [40, 41]. In an experimental model, the anabolic effect of ESWT on bone metabolism was detected by single-photon-emission computed tomography and microCT [19]. As this study did not show significant changes of bone microstructure parameters, the reason for the biomechanical improvement in ESWT groups seems not to be bony changes [21]. A possible reason may be the energy level used. In this study medium-energy level ESWT was applied. Moya et al. described, that best evidence for tendon disorders is provided for low- and medium-energy level ESWT. High-energy level ESWT seems to be effective in bone pathologies as well [11]. Van der Jagt et al. have shown the beneficial effect of ESWT on osteoporotic bone changes [42]. The effect and benefit of ESWT in the osteoporotic bone may be expectable in the humeral head after rotator cuff repair as well. Due to the increased risk of healing failure in patients with osteoporosis [6], further investigations focusing on this have already been initiated.

Earlier studies have also shown the impact of ESWT on tendon regeneration. Especially increased collagen synthesis by tenocytes as well as decreased expression of tendinopathy associated interleukins and matrix metalloproteases [14]. In muscle a single as well as repetitive ESWT resulted in improved blood flow [12]. Furthermore, oxygenation increase, a proliferative effect, and metabolic process activation have been described [43]. To simulate degenerative changes, repair of the tendon is performed after a 3-weeks period after supraspinatus tendon detachment. Buchmann et al. recommend this period due to the high self-healing potential in rats and the risk of fatty infiltration in humans [44]. In this study histological evaluation of muscle structure, nerve structures, and Collagen I and III showed no differences between the groups. HE and MSB staining indicate regenerated muscle- and tendon-tissue in all groups. This seems to be a confirmation of the chosen animal model and as a consequence a basis for further investigations. Histological analyses of CD31 staining provided the lead of increased blood vessel numbers in ESWT treated groups in the musculo-tendinous transition zone. Despite the low number of animals in histological evaluations and therefore missing possibilities of quantification, this seems to be the reason for the improved biomechanical results, as the tendon rupture in load-to-failure testing occurred mostly at this region of interest [21]. In particular, patients treated by arthroscopic double-row SSP tendon repair, suffer regularly from failure of the medial row with retears in the musculo-tendinous junction [45, 46]. Healing improvement by ESWT especially in this area may prevent from medial row failure. Therefore, further experimental studies focusing on this region and clinical studies are necessary and have already been initiated.

The use of rats with open growth-plates is possibly a limitation. For reasons of variability reduction and because of health-related problems in older rats, younger rats with comparable conditions were used according to earlier studies [4]. Another possible limitation is the small sample size for histological and immunohistochemical analysis. As primary focus was set on bone microstructure evaluations, rats were divided throughout examinations accordingly. Another limitation of this study is the missing of an interobserver reliability analysis for the imaging analysis.

In conclusion, bone microarchitecture changes are not responsible for previously described improved biomechanical results after shockwave treatment in rotator cuff repair in rodents. In contrast, immunohistochemical analysis showed neovascularization at the musculo-tendinous transition zone, an area susceptible to failure, in ESWT treated animals. Further studies focusing on neovascularization at the musculo-tendinous transition zone after ESWT are necessary to support these findings.
